# Evaluating the landscape and characteristics of endometriosis research published between 1990 and 2024

**DOI:** 10.1007/s00404-026-08516-x

**Published:** 2026-07-27

**Authors:** Shreya Mago, David Robert Grimes, Qian Feng, Ben W. Mol, Thomas Tapmeier

**Affiliations:** 1 Department of Obstetrics and Gynaecology, Monash University, Clayton, Melbourne, Australia; 2 Trinity College Dublin, Dublin, Ireland; 3 Department of Obstetrics and Gynaecology, Amsterdam University Medical Centre, Amsterdam, The Netherlands; 4 The Ritchie Centre, Hudson Institute of Medical Research, Clayton, Australia

**Keywords:** Meta-epidemiological study, Endometriosis, Power, Randomised-controlled trials

## Abstract

**Purpose:**

We aimed to assess the landscape of endometriosis randomised-controlled trials (RCTs) between 1990 and 2024 by characterising RCTs by management type, primary outcome, country of origin, trial registration, sample size and minimum detectable difference based upon standard RCT power targets of 80% and 90%.

**Methods:**

We identified all endometriosis RCTs from Dimensions.AI with an English abstract. Articles were examined for sample size, theme and primary outcome. Sample sizes were used to calculate minimum detectable effect size (MDES) of studies at 80% and 90% power.

**Results:**

We analysed 536 RCTs using ‘R’. 55.6% (*N* = 298) of studies investigated medical management of endometriosis, of which 177 investigated Gonadotrophin Releasing Hormone (GnRH) analogues. RCTs investigating complementary therapies rose from 0.847% (*N* = 1) in 1990–1999 to 28.7% (*N* = 31) of medical management RCTs in 2020–2024.

The US and China led publishing with 70 and 65 RCTs total, respectively.

Importantly, of 445 RCTs with at least one continuous outcome, we found that at 90% power, 55.7% (*N* = 248) of RCTs would only detect a standardised mean difference which was ‘large’ or greater.

**Conclusion:**

We noted several trends within endometriosis RCTs. In line with wider literature, China accounts for a growing proportion of RCTs. Although complementary therapy is increasingly recognised as a management option requiring further research, most medical management RCTs focussed on GnRH analogues as part of their treatment regime despite these being considered second-line management options. A high proportion of RCTs was underpowered to detect potentially clinically significant effects of intervention—potentially indicating significant resource wastage.

**Supplementary Information:**

The online version contains supplementary material available at https://doi.org/10.1007/s00404-026-08516-x.

## What does this study add to the clinical work?

**Table:** 

This study highlights the current research gaps within endometriosis RCTs through comparison of themes/management options historically investigated. Further, it brings to attention the under-powering of trials across endometriosis literature, suggesting that nearly half of endometriosis RCTs may not be sufficiently powered to detect a significant effect – this would indicate a significant wastage of resources within the field.	

## Introduction

Endometriosis is a multi-systematic, chronic inflammatory disorder characterised by the presence of endometrial-like tissue external to the uterus [1]. Despite its high prevalence, significant challenges remain in diagnosing [2] and managing the disease [3].

Clinical guidelines on endometriosis are limited by the lack of evidence, with even ‘strong’ recommendations having at best ‘moderate’ evidence underpinning them [3], likely due to historic underfunding [4]. Given the inability to provide strong recommendations based on the existing evidence [3], it is all the more imperative that investment into endometriosis research is carefully monitored to lead to the production of high-quality, clinically relevant research to maximise benefit from funding invested. This need is echoed within the creation of the Endometriosis Priority Setting Partnership [5], which attempts to maximise output from the limited research funding for endometriosis through identification of key questions considered most important to women with endometriosis and relevant practitioners. Further, the Australian *National Action Plan for Endometriosis* also lists high-quality, clinically translatable research focussing on disease prevention and cure as one of its core priorities [6].

The limited funding of endometriosis demands that resources be directed towards the most effective and impactful scientific practices**.** Achieving this begins with a clear understanding of the current research landscape, especially for randomised-controlled trials (RCTs). RCTs are widely considered the gold-standard for evaluating the efficacy and safety of medical interventions [7] but are resource-intensive [8]. It is thus essential that they are well designed to detect the effect they aim to identify. Sample size targets can be set by utilising an estimate of an effect size result considered clinically important based on prior studies and a target power, often set to 80 or 90% [9]. Despite this, underpowered trials make up a large portion of RCTs across biomedical literature [10, 11], with estimations that less than half of published RCTs meet their recruitment targets [12]. This results in reduced chances of detecting a true, and potentially clinically meaningful effect. Whether RCTs in endometriosis are adequately powered to detect clinically meaningful effects has not been previously studied.

One initiative endorsed to increase transparency of RCTs and reduce research waste is clinical trial registries [13]. These are aimed at reducing selective reporting by allowing open access to the methods and results of all commenced trials [13], not simply those which researchers or journals may find publishable [14]. From 2004, the International Committee of Medical Journal Editors required all authors submitting to member journals to register their trials in a registry meeting specific criteria [14], and in 2010, trial registration was added to the CONSORT guidelines for RCT reporting [15].

In this article, we characterise endometriosis RCTs according to theme and primary outcome of research, country of origin, sample size and minimum detectable difference based on standard RCT power targets of 80% and 90%.

## Methods

We performed a systematic review in the style of a meta-epidemiological study in the field of endometriosis reporting on RCTs published between 1990 and 2024. This review was registered on OSF retrospectively (30/05/2026).

### Identification of endometriosis studies

Dimensions.AI [16] was searched to create a database of all endometriosis studies published between January 1990 and the search date (4 March 2024).

An in-depth description of the Dimensions.AI search including search terms and database comparison is available in Supplementary File One.

### Identification of endometriosis RCTs

RCTs were identified in the formed database using a combination of PubMed study filters and text-mining for terms which suggested that the article was an RCT (see Supplementary File Two for strings of texts searched for).

Using the Dimensions ID of each article, full texts were searched for by SM and, if accessible, checked through to confirm all articles included were indeed RCTs pertaining to endometriosis. If full study texts were not available, abstracts of studies were used. If there was uncertainty regarding study classification, DRG was consulted.

### Analysing trends in RCTs

*‘*R’ programming was used for synthesis and analysis of all data and for creating figures, due to its ability to analyse large data sets and compute formulas.

#### Country of first author

‘Country of Author’ information provided by Dimensions.AI was used to analyse trends in publishing by countries over time. We used the country of the first author (as this is most commonly where the trial is completed) to rank countries by number of published RCTs.

#### Categorisation of RCTs by theme and primary outcome

RCT full texts were then manually checked by SM to categorise studies by theme. If the full text was not available, studies were classified based on their abstract. If there was uncertainty regarding theme or outcome, TT was consulted to aid in classification. TT reviewed 100 RCTs, so that inter-reviewer reliability could be assessed. Each RCT was marked as pertaining to ‘Management’, ‘Diagnosis’ or ‘Other’. Within ‘Management’, RCTs were allocated under; ‘Medical Management’, denoting any study pertaining to the administration of medication to endometriosis patients, ‘Procedural Management’, which denoted any study where procedural intervention such as surgery or minimally invasive procedures such as laser ablation or IVF was the primary intervention, ‘Alternative Medicine’, which denoted any management suggested as an alternative to conventional medical treatment such as homeopathy or Traditional Chinese Medicine, and finally ‘Complementary Therapy’, which included therapies suggested to accompany conventional management but were not medical or procedural, such as physiotherapy, or vitamin supplementation available without prescription. Please see Supplementary File Three for a full list of how each intervention was categorised. Although complementary and alternative medicine are frequently grouped together, these were classified separately in the present study to distinguish interventions intended to supplement conventional medical management from those positioned as substitutes.

The primary outcome of ‘Management’ RCTs was also noted and categorised as either; ‘symptom relief’ (which includes symptomology listed in the *Nature Reviews Disease Primer* for Endometriosis [17], except for fertility issues), ‘fertility’ (including all proxies utilised to assess for fertility such as live birth or clinical pregnancy and antral follicle count), ‘treatment complication’ (for studies comparing adverse effects of medication or procedural intervention), ‘quality of life (QoL)’ (which included studies using a QoL score or measuring markers relating to QoL such as work productivity), ‘extent of lesions/cure’ (which involved comparison of scores according to available endometriosis staging systems or size of cysts in the case of endometriomas or if it was self-described as having ‘cure’ as true primary outcome) or ‘other’ if it did not fit into the previous categories. If factors such as ‘fertility’ were being assessed to compare relative, known complications of differing intervention (e.g. surgical interventions causing negative impact on fertility outcomes), the primary outcome was denoted as treatment complications, as the primary purpose of the study was to reduce treatment complication as opposed to improving fertility. Some studies were allocated to multiple themes if there were multiple primary outcomes, for example, studies described as determining ‘safety and efficacy’ if containing primary outcomes pertaining to both were often designated under both ‘symptom relief’ and ‘treatment complications’.

#### Trial registration

As trial registry was added to the CONSORT guidelines in 2010, we identified the RCTs in our database published after 2010. We assessed all RCTs for which full text was available for reporting of trial registration within a WHO primary [18] or partner [19] registry and their registration number.

#### RCT sample size extraction and minimum detectable effect calculation

**Figure:**
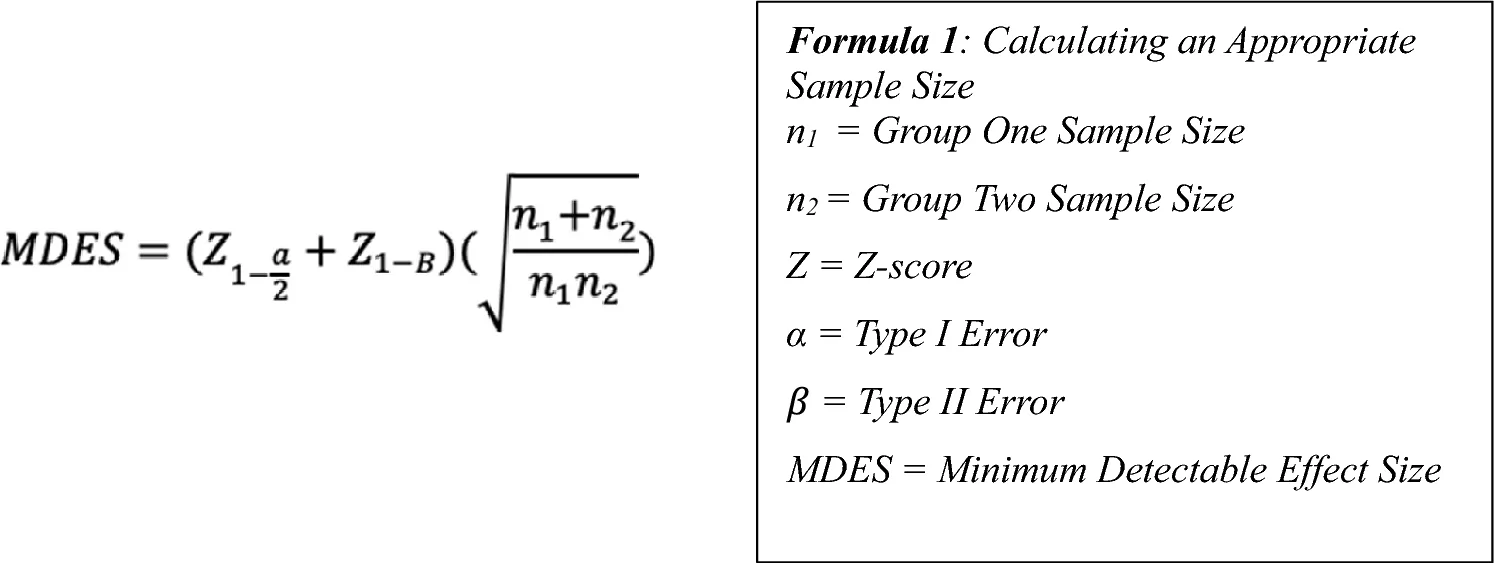

Following identification of RCTs, sample sizes were manually extracted from full texts and abstracts if full texts were not available. Sample sizes were recorded as total sample size and participants per group. Where provided, the participants recorded were the number of patients who commenced the intervention as this allows analysis of the power of studies at the outset of a trial. If the participants per group was not given, we recorded the total participants and the number of groups, and it was assumed there was an equal number of participants per group.

RCT outcomes were assessed to determine if they were binary or continuous values. Utilising the sample size extracted, the minimum detectable effect size (MDES) of RCTs with at least one continuous outcome was calculated using Formula 1 at 80% and 90% power. This formula used harmonic means to account for trials which had an uneven number of participants per group. We performed this equation through automated methods in ‘R’. If there were more than two groups, the code assumed that there were only two groups and identified the two numbers which would provide the ‘best’ MDES (smallest minimum effect) able to be detected by the study (See Supplementary File Four for formula derivation).

## Results

**Fig. 1:**
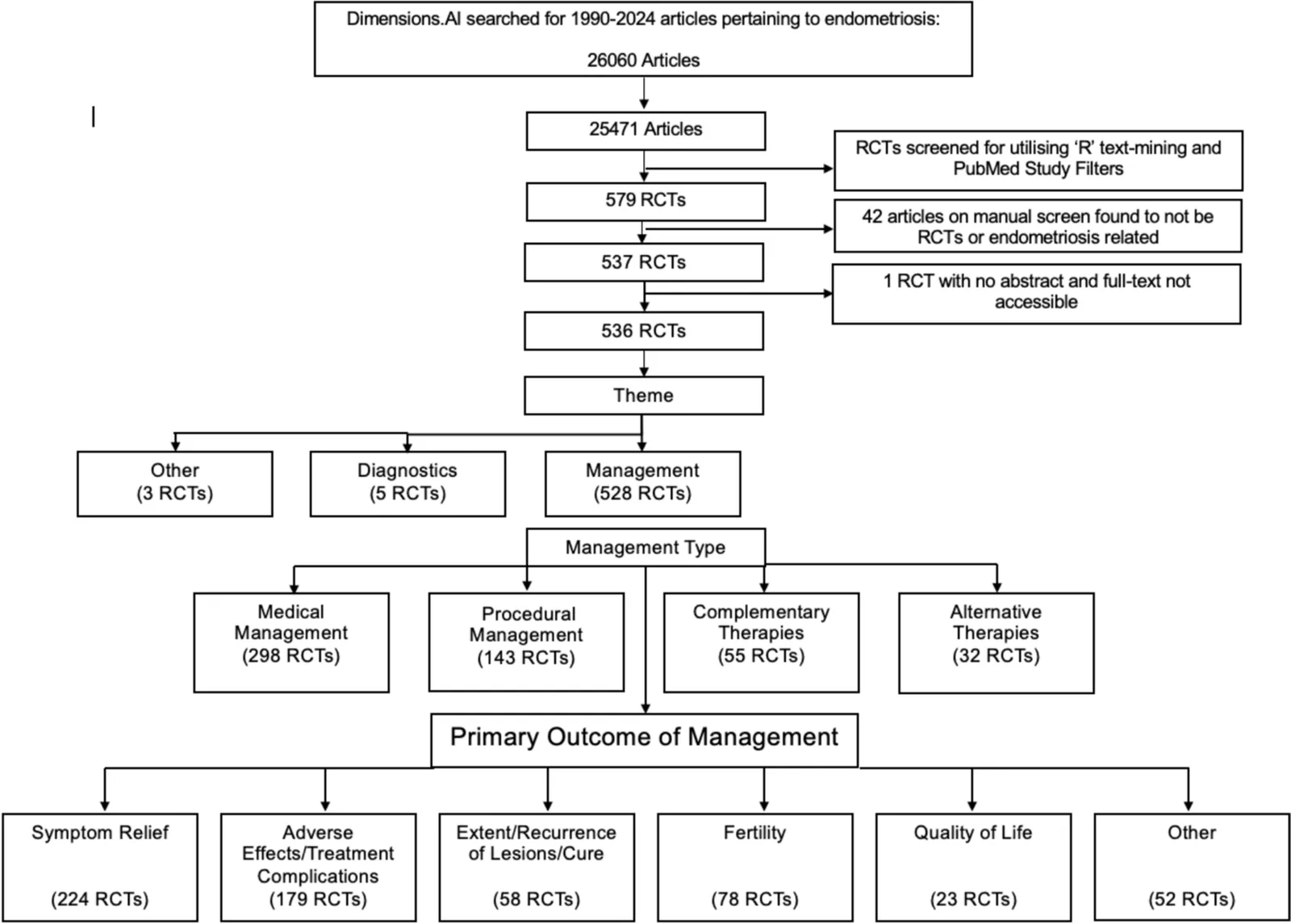
Flowchart of endometriosis RCT database refinement and thematic breakdown of trials

Overall, we identified 536 RCTs pertaining to endometriosis published between 1990 and 2024 (Fig. 1). Of these, we were able to access the full text of 464 RCTs, and the abstracts of the remaining 72 articles.

### Endometriosis RCTs by theme and primary outcome

We categorised the RCTs by theme and primary outcome (Fig. 1). The inter-rater reliability for theme was 94% and for primary outcome was 92%. We found that 98.5% (*N* = 528) of RCTs were categorised under ‘Management’ of endometriosis, five RCTs categorised as focussing on ‘Diagnostics’ of endometriosis and three were categorised as ‘Other’, focussing on themes such as prognostic prediction and pathophysiology.

In total, 56.4% (*N* = 298) of RCTs focussed on management were categorised under ‘Medical Management’ of endometriosis compared to 27.1% (*N* = 143) focussing on ‘Procedural Management’. Interestingly, we found that of RCTs focussing on ‘Medical Management’, 59.4% (177 RCTs of 298 focussing on ‘Medical Management’) used GnRH analogues in their treatment regime (this did not include RCTs which had used GnRH analogues as a control when trialling a new management option).

**Fig. 2:**
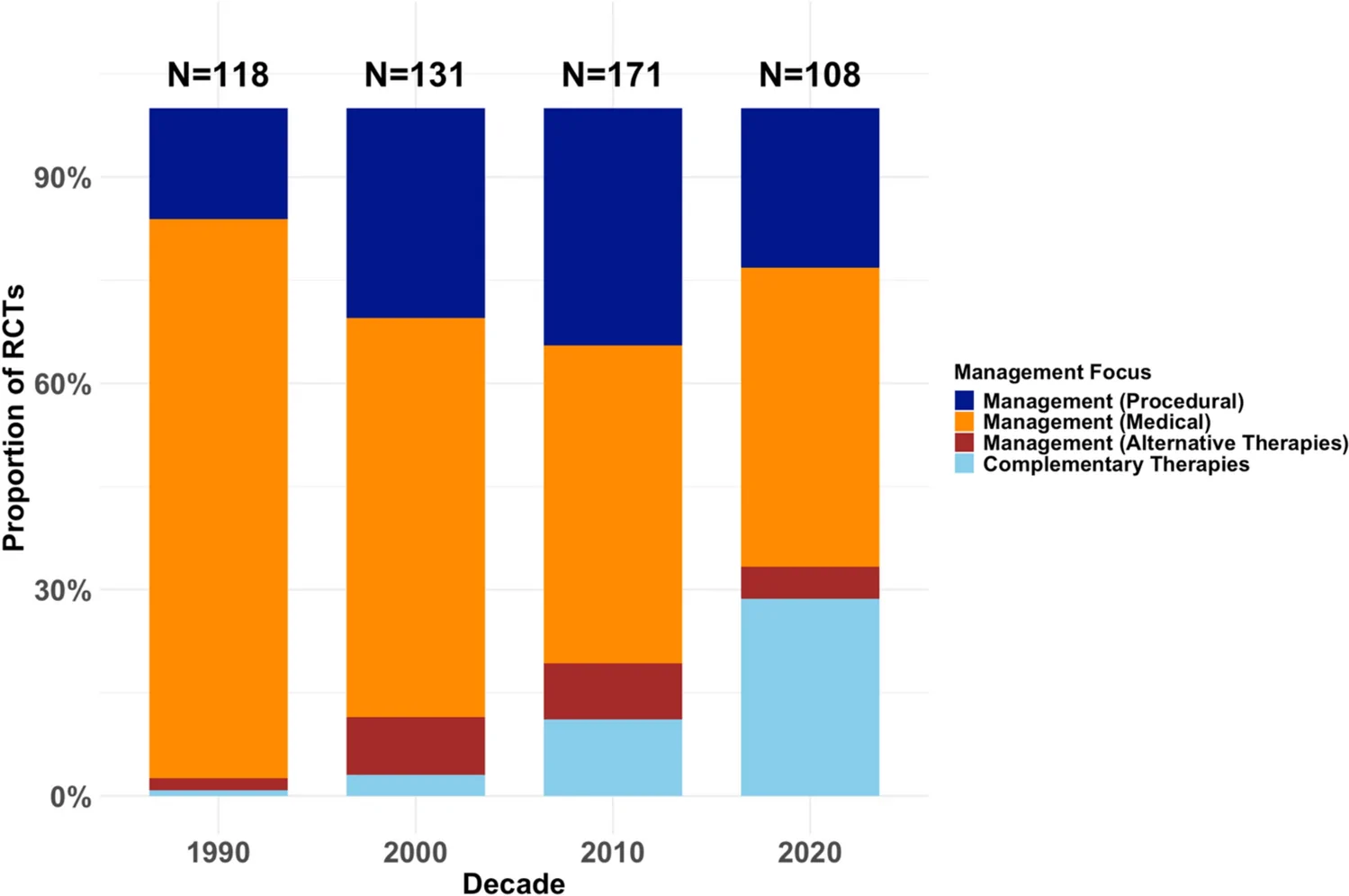
Change in management type of endometriosis investigated in RCTs over Time

There was a notable reduction in the number and proportion of studies focussing on ‘Medical Management’ since the 1990s, with 81.4% (*N* = 96) of RCTs published between 1990 and 1999 investigating ‘Medical Management’ compared to 58.0% (*N* = 76) in 2000–2009, 46.2% in 2010–2019 (*N* = 79) and 43.5% (*N* = 47) between 2020 and 2024 (Fig. 2). Also of note, we saw an increasing shift towards studies focussed on ‘Complementary Therapies’ in recent decades (Fig. 2), with 28.7% (*N* = 31) of RCTs categorised under ‘Complementary Therapy’ in 2020–2024, compared to only one of this study type being published between 1990 and 1999. ‘Procedural Management’ increased from just 16.1% (*N* = 19) of RCTs between 1990 and 1999 to 34.5% (*N* = 59) of RCTs published in 2010–2019 and 23.1% (*N* = 25) of RCTs in 2020–2024.

The 529 RCTs focussing on management were classified into six primary outcomes (Fig. 1). 70 of the RCTs had dual primary outcomes and therefore were categorised twice, 8 RCTs were categorised under 3 primary outcomes. The most common primary outcome was ‘Symptom Relief’, accounting for 42.4% (N = 224) of management RCTs, followed by ‘Treatment Complications’ accounting for 33.9% RCTs (N = 179). Additionally, 9.85% (N = 52) of RCTs were categorised as ‘Other’; these were mostly accounted for by studies focussing on changes to blood biomarkers or serum hormone levels caused by therapeutics.

### Endometriosis RCTs by country of publication

**Fig. 3:**
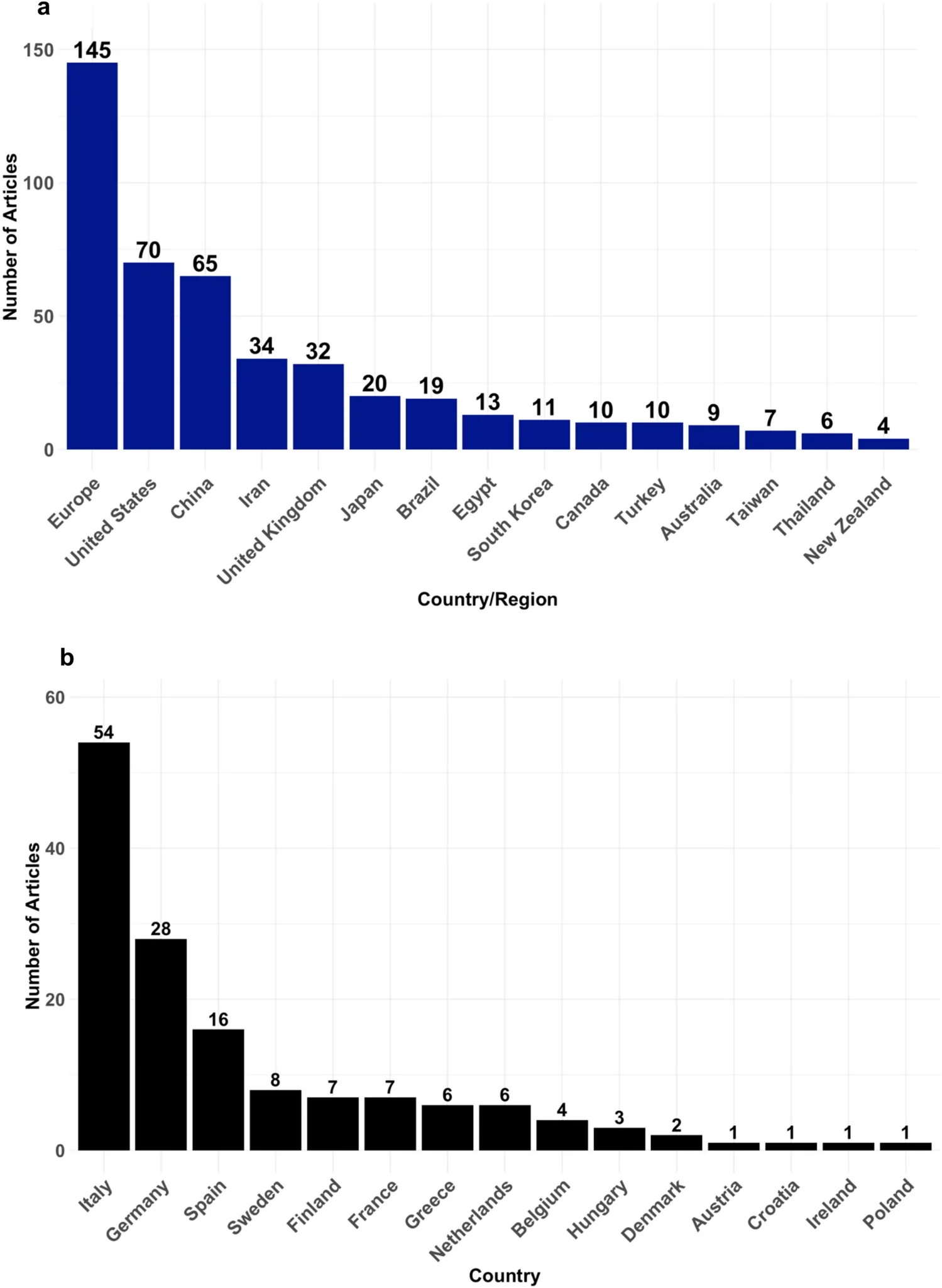
**a** Endometriosis randomised-controlled trials by country/region of first author (1990–2024); **b** European endometriosis randomised-controlled trial by country of first author (1990–2024)

We plotted RCTs by country of first author (Fig. 3). We found that the EU led overall with 145 RCTs (Fig. 3a), of which Italy accounted for 54, followed by Germany with 28 and Spain with 16 (Fig. 3b). Individually (Fig. 3a), the US and China led with 70 and 65 RCTs respectively, followed by Iran with 34 RCTs, the United Kingdom with 32 and Japan with 20.

### Distribution of sample sizes across endometriosis RCTs

**Fig. 4:**
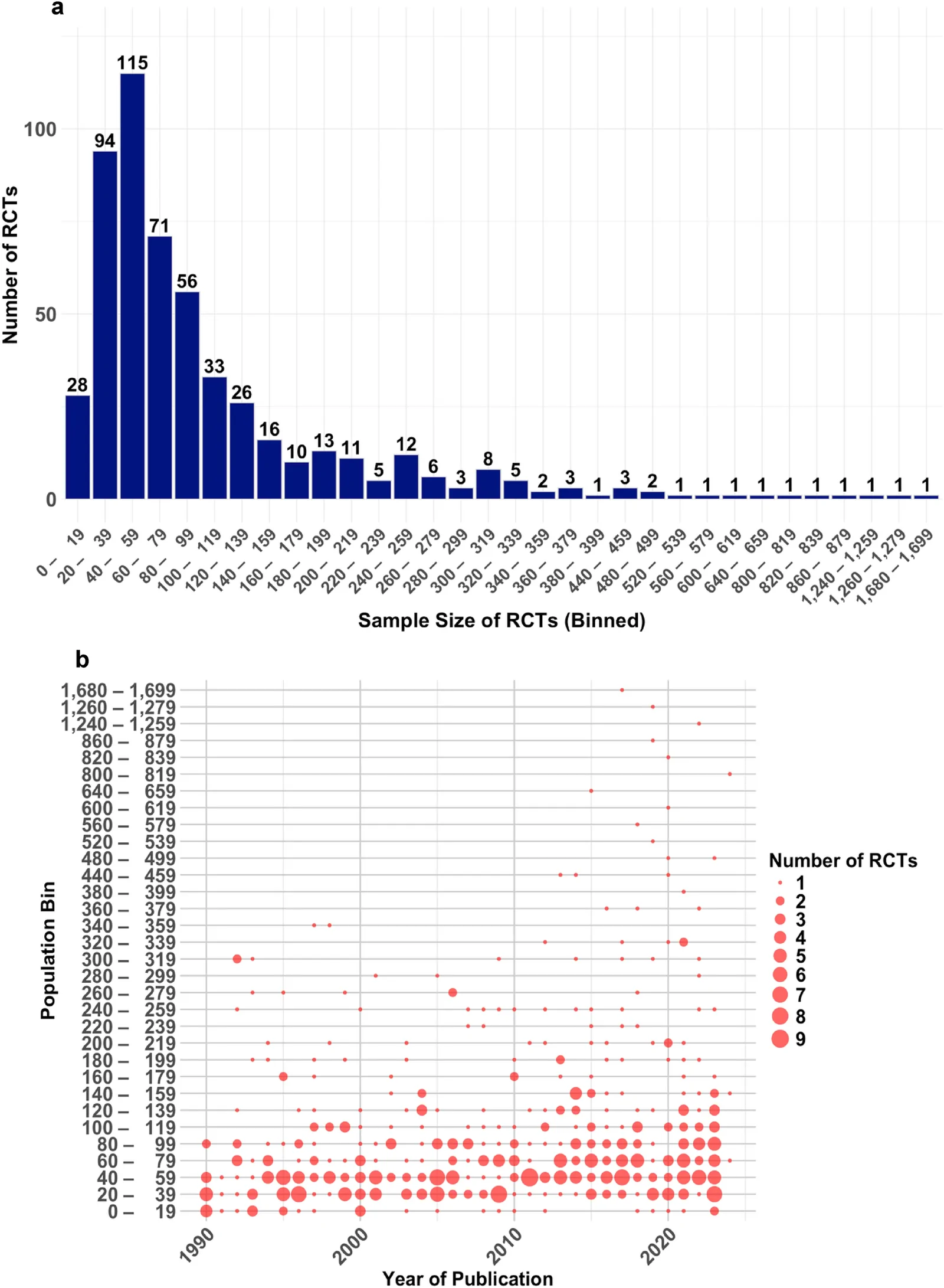
**a** Sample size of endometriosis randomised-controlled trials (1990–2024), binned; **b** change in endometriosis randomised-controlled trials over time (1990–2024)

Of the 536 RCTs, 533 provided a sample size, and were plotted in Fig. 4a, b. We were able to access the exact number per group in 497 of these 533 RCTs.

The most common sample size was between 40 and 60, which accounted for 21.6% of RCTs (*N* = 115) (Fig. 4a). Overall, RCTs with sample sizes under 100 accounted for 68.3% (*N* = 364) of RCTs.

There was a trend of increasing sample size with RCTs over time (Fig. 4b), with 23.5% (*N* = 28) of RCTs published between 1990 and 1999 having a sample size of greater than 100, compared to 41.3% (*N* = 45) in 2020–2024.

### Minimum detectable difference across endometriosis RCTs

**Fig. 5:**
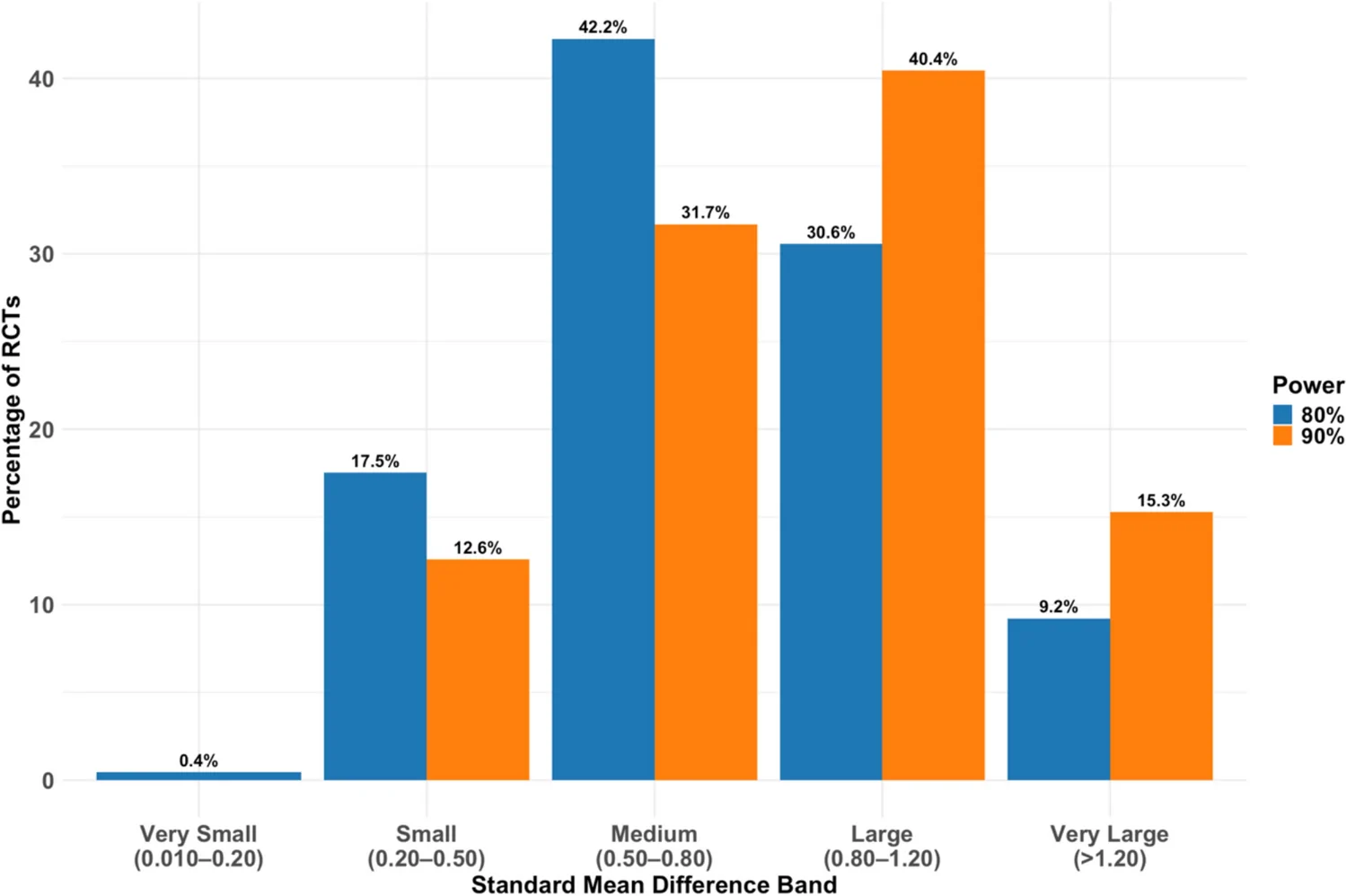
Minimum detectable difference in endometriosis randomised-controlled trials (1990–2024) at 80% and 90% power

445 RCTs had at least one continuous outcome. We found that at 80% power, 39.8% of RCTs (*N* = 177) would not have been able to detect an effect unless the size of the standardised mean effect (SMD) considered ‘large’ or greater (Fig. 5). If aiming for 90% power, 55.7% (*N* = 248) of RCTs would only be able to detect an SMD which was ‘large’ or greater. Further, at 90% power, 15.3% of RCTs (*N* = 68) would have only been able to detect ‘very large’-sized SMDs or greater.

### Trial registration

**Fig. 6:**
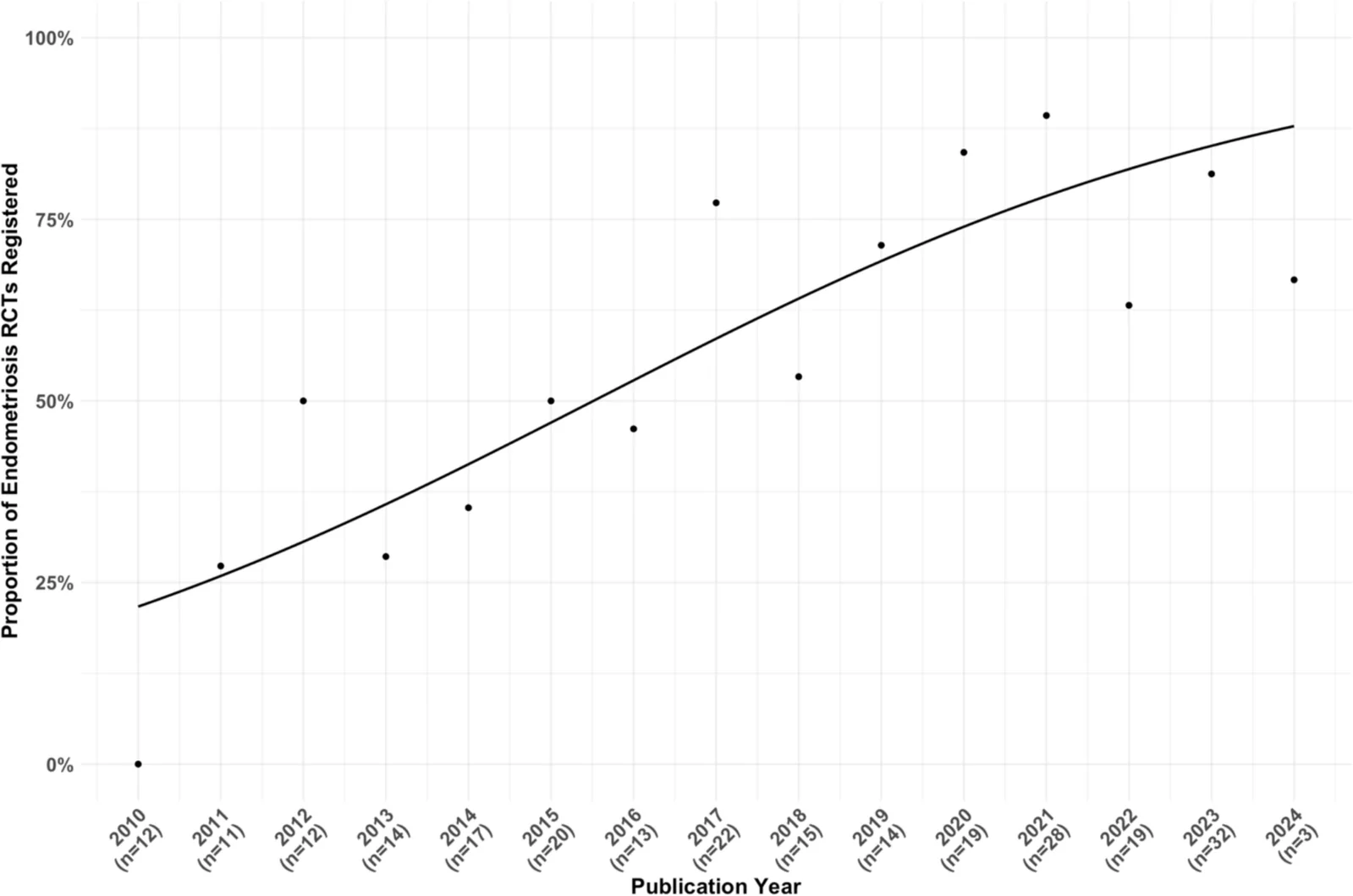
Proportion of endometriosis RCTs registered within trial registries over time; ‘n’ refers to the number of endometriosis RCTs published each year

Of 251 RCTs published from 2010 onwards for which full text was available, we identified 59.9% (151 RCTs) which reported registering their trial in a WHO primary or partner registry. There was a clear trend of increase in proportions of RCTs registering their trial over time (see Fig. 6), rising from no trials published in 2010 to a high of 89.3% of 28 RCTs published in 2021.

## Discussion

Here we evaluated the landscape of endometriosis randomised-controlled trials between 1990 and 2024 through analysis of the changes in sample size, management and primary outcome investigated through RCTs. We found a high proportion of studies focussing on medical management in early years (1990–1999), with increasing focus on procedural options and complementary therapies in later years. Amongst RCTs, Europe led with the highest total number of RCTs published, followed by the US and then China. Further, we found that approximately half of endometriosis RCTs were insufficiently powered to detect harm or treatment effect unless minimum standardised difference between trials groups was ‘large’ or greater.

The presence of China within the top three publishing regions of endometriosis RCTs follows trends seen in the wider biomedical literature. China’s spending on research has increased by 400-fold in the past 40 years, and the country now invests more into research than the whole of Europe and is approaching parity with the US [20]. Despite this significant, and rapid, surge in research output, it appears that the quality of research emerging from China is high and steadily improving, with steadily increasing Category Normalised Citation Impact [20] and a ranking of second (at the time of writing) amongst *Nature’s Index of Health Sciences* [21].

Amongst RCTs, we saw a considerable increase in the proportion of RCTs focussing on complementary therapies in recent decades. This is in line with other literature suggesting high levels of interest in non-pharmacological management amongst endometriosis patients [22, 23], likely due to dissatisfaction with available medical management options. Further, it was noted that the majority of RCTs investigating medical management of endometriosis included GnRH analogues within their treatment regime, despite these medications considered second line for endometriosis-related pain [3] due to their considerable side effect profile. This suggests a need to begin focussing our RCT resources on other, more tolerable management options, which may also explain the increasing research into surgical management over time.

RCTs are considered the gold standard of research design [7] and one of the final steps in the development of clinical interventions [8]. MDES of RCTs indicates the minimum standardised mean difference which needs to be present between treatment groups to be detected as statistically significant at a given sample size and target power of the RCT [24]. Context is recommended in determining the target MDES for a trial, utilising similar trials and cost of intervention to determine what may be considered an expected and clinically important effect [24]. For context, the SMD for bone mineral density in patients receiving danazol or gestrinone versus GnRH analogues in a systematic review by Farmer et al. [25] was 1.17, indicating a large effect as per Cohen’s classification. This result was statistically significant, reflecting a clear difference between the two treatment groups. In contrast, the impact of gel agents in reducing post-operative adhesions had an SMD of 0.24 (small effect under standardised classifications); however, this was considered uncertain by Ahmad et al. [26] as it did not reach statistical significance. It is possible that with a larger sample size this result may have reached statistical significance, and the failure to detect a significant effect for this small SMD may therefore reflect insufficient statistical power rather than a true absence of effect. Using the distribution of sample sizes across endometriosis RCTs, we found that due to insufficient sample size, close to half of our RCTs would not have been able to detect a treatment or harm effect at 90% power, unless the standardised difference between groups was ‘large’ or ‘very large’, based upon standard classifications of standardised mean difference. Given the current lack of satisfactory endometriosis management options [3] and severe symptomology experienced by those affected [2], an intervention resulting in ‘medium’ or even ‘small’ effect, especially if accompanied by low cost or minimal adverse effects, may be of clinical relevance. This is particularly the case for patients experiencing significant symptomology. This is considerably relevant in the context of an increasing number of RCTs focussing on complementary therapies as found in this study, which are often low cost compared to the conventional surgical and medical management. The clinical consequence of this finding is that treatment which may substantially improve patient quality of life may not be identified by a large proportion of current endometriosis RCTs due to inadequate design. Given the significant challenges already faced by endometriosis research given the complex nature of the disease and the historical lack of funding [4], it is imperative research efforts and scarce resources are not wasted due to underpowering of trials, thereby adding an additional barrier to the finding of effective endometriosis management options. Further, although average sample sizes have improved over time, small sample RCTs continue to be present, suggesting that these potential clinical consequences are ongoing. It is likely this is due to ongoing challenges in recruitment, which may be aided by the increased use of multi-centre trials [27].

Reassuringly in regard to efforts in tackling research waste, we found a steadily increasing trend in the proportion of RCTs which were registered within a trial registry, consistent with other specialties [28, 29]. However, a study by Tan et al. [30] found that RCTs across medicine published in 2017 had a trial registration rate of 95%, suggesting that endometriosis may lag behind the broader medical field. Further, as emphasised in the 2025 CONSORT guidelines [31], endometriosis researchers must continue to strive towards transparency within scientific reporting, including through registration of RCTs.

A strength of this study was coverage of a significant quantity of research. Dimensions.AI has been noted by multiple studies to have high overlap in articles and journals with longer established databases such as Scopus [32], PubMed [33] and WoS [34], whilst also covering a large number of sources not covered by these databases [34, 35]. As the study aimed to analyse the landscape of *all* endometriosis RCTs undertaken between 1990 and 2024, this extensive coverage of studies should better allow comprehensive analysis. Further, utilisation of multiple methods, including PubMed study classifications (which have previously been validated [36]) and text-mining, allowed for enhanced accuracy in identification of RCTs, which were then able to be verified with manual checking. Further, no other study to our knowledge has previously attempted to characterise endometriosis RCTs or to assess their ability to detect clinical effect—particularly important in the context of advocating for increasing funding and research towards endometriosis. Finally, this study is the first within our knowledge to assess the rate of trial registration amongst endometriosis RCT.

This study also had limitations. Several studies indexed as RCTs by PubMed study classifications or through text-mining analysis of abstracts were not part of Open Access journals, and thus, their full texts were inaccessible to us, as they would be to a significant number of readers. However, much of the information required such as sample size and theme was easily obtained from abstracts. Further, we included publications potentially from the same trial, with separate publications reporting on different outcomes. This was because Publication IDs and order of authors may differ between studies, and many earlier trials did not include registration numbers. These factors made it difficult to identify if two articles were pertaining to the same trial. Hence, we included all RCT studies to ensure that we included all outcomes and management options investigated by RCTs. Further, the rate of trial registry did not include whether trials had been prospectively or retrospectively registered—something which should be reviewed in the future. Finally, we used the country of first author to plot country of publication as we assumed that this was where trials were performed; however, this may not be true across all published RCTs. As the aim of the study was to obtain a trend of publishing output by country, this should not have significant impact on conclusions drawn.

## Conclusion

We analysed endometriosis RCTs published between 1990 and 2024. Geographically, we saw China emerge as one of the highest publishers of endometriosis RCTs, in line with wider biomedical literature. Amongst RCTs focussed on management, we found a substantial volume of literature dedicated to GnRH analogues, despite these being considered second-line management options due to significant adverse effects, and an increasing number of studies focussing on complementary therapy in the most recent period of 2020–2024. Unfortunately, we found that the majority of RCTs would only be able to detect treatment effect if these effects were considered ‘large’ or greater at 90% power. Inadequate sample sizes and therefore underpowering of RCTs may result in missed clinically important and viable management options—a severe clinical consequence in the context of the substantial disease burden experienced by endometriosis patients.

## Supplementary Information

Below is the link to the electronic supplementary material.

Supplementary file1 (DOCX 32 KB)

Supplementary file2 (XLSX 778 KB)

## Data Availability

All data supporting the findings of this study can be reproduced using the information available within the paper and its Supplementary Materials.
