# Supplementary material for: Evaluating the landscape and characteristics of endometriosis research published between 1990 and 2024
Source: Arch Gynecol Obstet. 2026 Jul 27;313(1):299. doi: 10.1007/s00404-026-08516-x (PMC13687228; doi:10.1007/s00404-026-08516-x)
Supplement: Supplementary file 1 [file 404_2026_8516_MOESM1_ESM.docx]

**Supplementary File 1**

**Identification of Relevant Studies**

**Search Strategy**

We created a database to identify all endometriosis research published between 1 January 1990 and search date (4 March 2024). We searched four online databases – Dimensions.AI (https://www.dimensions.ai/), PubMed, Web of Science and Scopus. The search strategy was developed using previous systematic reviews^[1,2]^ pertaining to endometriosis with listed search strategies as a basis, to ensure a maximum number of relevant articles were identified. The search was further refined through continuous manual verification, with 100 articles randomly selected to ensure relevance until a search strategy was finalised. The final search strategy yielded less than 5% results irrelevant to endometriosis upon manual verification in all databases – comparable to the search precision of other databases.

| **Number of Endometriosis Articles featured on differing databases and comparison of database features** | | |
| --- | --- | --- |
| **Database** | **Number of Results** | **Features** |
| Pubmed | 24926 | - Able to filter by study type (e.g. case studies, RCTs, observational studies, systematic reviews, literature review) - Not all studies included within filter |
| Scopus | 24473 | - Able to filter articles and reviews - Geographic and sponsorship information of studies |
| Dimensions.AI | 26061 | - Able to filter articles and reviews - Geographic and sponsorship information of studies |
| Web of Science | 19366 | - Able to filter articles and reviews - Geographic and sponsorship information of studies |

### **Search Strategy**

*Dimensions:*

(((((((mesh_terms:Endometriosis) OR (mesh_terms:(Endometrioma ))) OR (mesh_terms:(Adenomyosis ))) OR (title:Endometriosis)) OR (title:Endometrioma)) OR

(title:Adenomyosis)) NOT (title:Adenomyomatosis)) NOT (title:nonendometriosis)

su

*PubMed*

*(((((((endometriosis[MeSH Terms]) OR (endometrioma[MeSH Terms])) OR (adenomyosis[MeSH Terms])) OR (endometrioma[Title])) OR (endometriosis[Title])) OR (adenomyosis[Title])) NOT (adenomyomatosis[Title])) NOT (nonendometriosis[Title])*

WoS

(((((((TI=(Endometriosis )) OR TI=(Endometrioma)) OR TI=(Adenomyosis)) OR AK=(Endometriosis)) OR AK=(Endometrioma)) OR AK=(Adenomyosis)) NOT ALL=(Adenomyomatosis)) NOT TI=(nonendometriosis)

Scopus

TITLE ( endometriosis ) OR TITLE ( endometrioma ) OR TITLE ( adenomyosis ) OR AUTHKEY ( endometriosis ) OR AUTHKEY ( endometrioma ) OR AUTHKEY ( adenomyosis ) AND NOT TITLE ( adenomyomatosis ) AND NOT TITLE ( nonendometriosis )

**Justification for use of Dimensions.AI**

Due to the high likelihood of duplicates unlikely to be identified through automated methods, we chose not to combine databases given the potential introduction of inaccuracy to our results analysis. Hence, Dimensions.AI was selected due to its significant coverage of journals and articles.

We found that Dimensions.AI search results included 85.7% of PubMed results whilst also containing additional articles not yielded by the PubMed search.

Dimensions.AI’s significant coverage of journals and articles has been validated in multiple studies. Dimensions.AI covers the vast majority of journals^[3]^ and articles^[4]^ covered by Scopus and Web of Science (WoS) – covering 96.42% of Scopus and 96.61% of WoS - whilst also covering an extensive number of additional journals. Further, it has been shown to have extensive coverage of PubMed – covering 99.98% of documents in the 2020 PubMed Baseline^[5]^.

We created a database to identify all endometriosis research published between 1 January 1990 and search date (4 March 2024). We searched Dimensions.AI with a search strategy developed through adaptation of search strategies from previous systematic reviews^[1,2]^ pertaining to endometriosis. The search was further refined through continuous manual verification of 100 articles, with the final search strategy yielding less than 5% results irrelevant to endometriosis.

**References**

1.	Kvaskoff M, Mahamat-Saleh Y, Farland LV, et al. Endometriosis and cancer: a systematic review and meta-analysis. *Human Reproduction Update*. 2021;27(2):393-420. doi:10.1093/humupd/dmaa045

2.	Bafort C, Beebeejaun Y, Tomassetti C, Bosteels J, Duffy JM. Laparoscopic surgery for endometriosis. *Cochrane Database of Systematic Reviews*. 2020;2020(10)doi:10.1002/14651858.cd011031.pub3

3.	Singh VK, Singh P, Karmakar M, Leta J, Mayr P. The journal coverage of Web of Science, Scopus and Dimensions: A comparative analysis. *Scientometrics*. 2021;126(6):5113-5142. doi:10.1007/s11192-021-03948-5

4.	Visser M, Van Eck NJ, Waltman L. Large-scale comparison of bibliographic data sources: Scopus, Web of Science, Dimensions, Crossref, and Microsoft Academic. *Quantitative Science Studies*. 2021;2(1):20-41. doi:10.1162/qss_a_00112

5.	Liang Z, Mao J, Lu K, Li G. Finding citations for PubMed: a large-scale comparison between five freely available bibliographic data sources. *Scientometrics*. 2021;126(12):9519-9542. doi:10.1007/s11192-021-04191-8

### **Supplementary File 2**

### **Identifying RCTs using R**

RCT_pattern <- "randomised_trial|randomised_clinical_trial|randomized_clinical_trial|

randomised_controlled_trial|randomised_control_trial|randomized_trial|randomized_controlled_trial|

randomized_control_trial|randomly_allocated|\\brct\\b|placebo-controlled|placebo-group|placebo_controlled|

placebo_group|single_blinded|double_blinded|double-blinded|single-blinded"

Although PubMed was not used to form the results database, as PubMed has a function to filter by publication type (e.g. RCTs, Systematic Reviews etc.) which has been previously validated^1,2^ and given the large overlap of studies between PubMed and Dimensions results, the PubMed ‘publication type’ filter was used to assist in sorting the refined database into study type to identify RCTs

From here, to identify additional RCTs, titles were checked using Microsoft Excel for ‘RCT’ or ‘randomised’ or ‘randomized’ and such titles were screened through and added to the PubMed RCT results.

Finally, for studies which had not been classified by PubMed or did not overlap with the PubMed results, ‘R’ programming was used to identify additional RCTs.

**Supplementary File 3 – Classification of Studies by Management Type**

If there was a combination of surgical and medical intervention, it was categorised as whichever was not constant between the two groups (e.g. if both groups received surgery first but then received different medical management following)

Medical Management

- Hormonal therapy (including implant devices such as IUDs)
- GnRH Analogues
- Aromatase Inhibitors
- Hormone Replacement Therapy
- ASP1707
- Mifepristone
- Danazole
- Genistrone
- SERMs
- Clarithromycin
- Atosiban
- Perfenidone
- Corticosteroids
- Oxytocin
- BAY 86-5047
- Pentoxifylline
- NSAIDs
- Pioglitazone
- Statins
- Ullipristal Acetate
- BAY 1158061
- Infliximab

Procedural

- Surgery
- HIFU Ablation
- Artificial Insemination
  - E.g. fallopian tube sperm perfusion (FSP) with 4 mL of inseminate and intrauterine tuboperitoneal insemination (IUTPI)
- Lignocaine pertubation
- IVF
- IUI
- ICSI
- Changes to IVF/ICSI protocol
- Changes to immediate pre- or post-op management (e.g. PCA for post-op pain for 6 hours post-op, ketorolac immediately prior to anaesthesia)
- Changes to intraoperative management (e.g. injecting vasopressin into mesovarium, intraperitoneal ropivacaine to reduce post-op pain)
- Gel infusion sonography
- Change in surgical protocol (e.g. use of fast track)
- Intraoperative products to reduce adhesions
- USS guided endometrioma aspiration
- Laser laporoscopic evaporation
- Tubal flushing
- USS guided interleukin-2 injection
- hysteroscopic-guided pertubal diluted bupivacaine infusion

Complementary

- Supplements (Vitamin C, silymarin, vitamin D, curcumin, Lactobacillus gasseri (probiotic), vitamain E, garlic supplementation, antioxidant supplementation, melatonin, “Wobenzym”
- Transcranial direct stimulation
- Resveratrol
- Electrotherapy
- Muscular relaxation
- Sensate focus
- Mindful based intervention
- Educational intervention
- Selfcare counselling
- Dietary Changes
- Natural extracts
- Manual therapy
- Supervised Exercise Program
- Yoga
- Pulsed laser therapy
- Psychotherapy
- Physiotherapy
- Virtual Reality Simulations (Immersive Digital Therapeutic – Endocare)
- Acupuncture
- TENS
- Hypnotherapy
- Omega-3 fatty acids
- Vagal Afferent Nerve Stimulation

Alternative

- Homeopathy
- Traditional Chinese Medicine

**Supplementary File 4**

**Effect Size derivation**

The standardised mean difference (SMD, $\theta$) is given by the difference in mean of both groups, divided by their standard deviation, or

$$\theta=\frac{\mu_{1}-\mu_{2}}{\sigma}.$$

If the enrolment ratio between two groups is given by $k= \frac{n_{2}}{n_{1}}$ , then the standard formula for calculating sample size per group is

$$n_{1}=\sigma^{2}\left( 1+\frac{1}{k} \right)\left( \frac{Z_{1-\frac{\alpha}{2}}+Z_{1-\beta}}{\mu_{1}-\mu_{2}} \right)^{2}=\left( 1+\frac{1}{k} \right)\left( \frac{Z_{1-\frac{\alpha}{2}}+Z_{1-\beta}}{\theta} \right)^{2}.$$

Similarly, the sample size of the second group is given by $n_{2}=kn_{1}.$ To find the resolvable SMD given just group size, we can reverse-engineer the standard equation and with a known $n_{1}$ and $n_{2}$, calculate the SMD. Re-arranging for $n_{1}$ equation and substituting the value of $k$ gives us the square of the SMD, namely

$$\theta^{2}=\left( \frac{n_{1}+n_{2}}{{n_{1}n}_{2}} \right)\left( Z_{1-\frac{\alpha}{2}}+Z_{1-\beta} \right)^{2}.$$

Thus, given group sizes $n_{1}$ and $n_{2}$, the resolvable SMD is thus

$$\therefore\theta_{Resolvable}=\left( \sqrt{\frac{n_{1}+n_{2}}{{n_{1}n}_{2}}} \right)\left( Z_{1-\frac{\alpha}{2}}+Z_{1-\beta} \right).$$
